# Clozapine and the Risk of Severe COVID-19: A Retrospective Cohort Study

**DOI:** 10.1192/j.eurpsy.2024.558

**Published:** 2024-08-27

**Authors:** I. Yaich, A. Touiti, C. Ben Said, N. Bram

**Affiliations:** ^1^Forensic Psychiatry Departement, Razi Hospital, La Manouba; ^2^Faculty of Medecine of Tunis, Tunis El Manar University, Tunis, Tunisia

## Abstract

**Introduction:**

Clozapine is the standard treatment for managing treatment-resistant schizophrenia (TRS). However, concerns arise due to potential hematologic side effects, such as agranulocytosis, especially during the COVID-19 pandemic.

**Objectives:**

This study aims to investigate the association between clozapine treatment and an increased risk of severe COVID-19 infection in patients with TRS.

**Methods:**

A retrospective study reviewed clinical records of forensic patients with TRS from 2020 to 2022 at Razi Hospital’s forensic psychiatry department in Tunisia. Twenty-five patients, including 18 on clozapine treatment, were included.

**Results:**

All patients were male, with an average age of 39.7 years. Twenty-three patients received at least one vaccine dose. Twenty-two patients contracted COVID-19. Among those treated with clozapine, two required intensive care unit admission and oxygen therapy without intubation. Clozapine treatment remained uninterrupted, with no dose escalation during infection episodes. Lymphopenia was the most commonly reported hematologic abnormality.

**Conclusions:**

While there may be an association between clozapine use and an increased risk of COVID-19 infection, no clear correlation with infection severity and antipsychotic treatment was established in this study. Further research is needed to explore this potential association comprehensively.

**Disclosure of Interest:**

None Declared

